# Gratuitous Services to Clergymen

**Published:** 1880-01

**Authors:** 


### EDITORIAL.

### GRATUITOUS SERVICES TO CLERGYMEN.

SEVERAL of the medical periodicals, *The Philadelphia Medical Times* and *The Medical Record* among the number, have been discussing, of late the question why clergymen receive gratuitous attendance from the medical profession. The special tax thus imposed upon physicians and also upon druggists, who are fellow-sufferers from like impositions, has long ago given rise to serious doubts as to its justice, from many of our most enlightened men. Our esteemed contemporaries wisely conclude that this custom should be abolished, and clergymen placed on the same level with all other classes and professions requiring and seeking medical services. It is true that many clergymen are poorly and inadequately compensated for the ability, erudition and zeal they bring to their sacred calling. To all such, the medical profession is only too willing to lend the helping hand, and to show their practical appreciation of self-sacrificing labors in the interests of humanity, by responding to their calls. But in cases of clergymen who receive annually a salary of two or three or five thousand dollars, more than the majority of physicians can by any possibility earn, we fail to see the justice or the wisdom of this exhibition of purely American philanthropy. If the profession estimate the good-will and favor of the clergy to be of assistance in building up a practice, hoping, figuratively speaking, that the sheep will follow their shepherd, they are too often grievously disappointed. *The Record* well says that practice obtained by such means "is not worth a great deal, especially if the unfortunate practitioner has the representatives of all the denominations in his village on his list. Even then it is quite likely, that his distinguished patient may recommend some quack to his parishioners, especially if that individual has made a fortune, and is a prominent pewholder in the church. The good influence which might be created in behalf of legitimate medicine is thrown into another channel, and charlatanry is endorsed not only in religious papers, by widely circulated certificates of remarkable cures, but even in the pulpit itself." This confirms our own experience that clergymen, strange and inconsistent as it may appear in view of their boasted intelligence, are often the most willing dupes of the basest medical imposters.

We fail to find any valid reason for these gratuitous services. The majority of the profession are members of or attendants on some of the religious bodies, and share in the expense of their maintenance, paying their quota of the minister's salary. On what principle, certainly not of equity and justice, are they expected to dispense to the clergy, without compensation, the skil! and knowledge, acquired at a large expenditure of money, and of patient labor and effort, while the mechanic and laboring man enjoy no such exemption?

Besides, it is a fact that services, thus freely and generously rendered, are but poorly appreciated by our clerical patrons. We do not approve of pauperization, whether among the genteel and educated, or among the ignorant and vulgar. No class of men, professional or otherwise, are beyond the reach of such demoralizing influences. We approve of a just compensation for the clergy, as well as for lawyers, teachers and mechanics, and we especially endorse the apostolic injunction that the clergy should "owe no man anything," not even their doctor, "but to love one another," in which category we trust the profession of medicine, always abundant in good works in ameliorating the sufferings to which flesh is heir, may be included.

